# openSSS: an open-source implementation of scatter estimation for 3D TOF-PET

**DOI:** 10.1186/s40658-025-00730-x

**Published:** 2025-02-28

**Authors:** Rodrigo José Santo, André Salomon, Hugo W. A. M. de Jong, Simon Stute, Thibaut Merlin, Casper Beijst

**Affiliations:** 1 Department of Radiotherapy, Imaging & Oncology Division, UMC Utrecht, Utrecht, The Netherlands; 2 Philips Research Europe, Eindhoven, The Netherlands; 3 Department of Radiology & Nuclear Medicine, UMC Utrecht, Utrecht, The Netherlands; 4 Nuclear Medicine, CHU de Nantes, Nantes, France; 5 CRCINA, INSERM, CNRS, Université d’Angers, Université de Nantes, Nantes, France; 6 LaTIM, INSERM, UMR 1101, University of Brest, Brest, France

**Keywords:** Scatter correction, Quantitative PET, TOF-PET, Open-source software

## Abstract

**Background:**

Scatter correction is essential for quantitative and accurate time-of-flight (TOF) PET imaging. It is implemented by an accurate scatter estimation algorithm, to calculate the statistical distribution of scattered photons among the measured coincidences. However, to our knowledge, scatter estimation algorithms that account for TOF and that are compatible with custom geometries are not available in open-source reconstruction libraries, such as CASToR and STIR. To this end, we have developed an open-source implementation of the TOF-aware single-scatter-simulation (SSS) algorithm: openSSS.

**Results:**

openSSS is validated on NEMA phantoms and patient data, for three PET geometries, compared to Monte-Carlo simulations and two proprietary vendor-specific reconstruction platforms. The reconstructed images have similar contrast recovery and background variability, deviating by up to 3.7%-point on contrast recovery and 1.8 on background variability and looking visually similar.

**Conclusion:**

We have developed and validated an open-source scatter estimation library to complement reconstruction frameworks. By enabling vendor-independent clinical-grade reconstructions on custom scanner geometries, openSSS represents a crucial step in transparent research on quantitative PET and novel PET scanner designs.

## Background

Positron emission tomography (PET) images are obtained through tomographic reconstruction of acquired projection data, relying on the accurate measurement of the two annihilation photons. These particles are emitted in opposite directions, but their trajectory may change due to scatter in the patient. This results in a misplacement of the measured line-of-response (LOR) and, consequently, reduced image quality.

Scattering can be elastic (Rayleigh) or inelastic (Compton), with the probability and differential cross-section being dependent on the photon’s energy. In the human body, Compton scattering is the most dominant interaction for annihilation photons (511 keV) and it is characterized by interactions with outer-shell electrons, resulting in ejection of the electron and energy change of the photon. Rayleigh scattering happens for interactions with tightly-bound electrons, with no energy change for the incident photon.

Quantitative TOF-PET relies on accurate scatter correction, as the scatter fraction typically ranges from 30 to 50% of the true detected coincidences in typical clinical acquisitions. Scatter correction is realized by an accurate scatter estimation algorithm, since it is not possible to filter out the scatter effect for each measured coincidence. Instead, only statistical correction during reconstruction is possible, based on the estimation of the distribution of scattered photons. Without scatter correction, image quality is degraded by loss of contrast and inaccurate quantification.

Scatter correction is typically performed using the model-based time-of-flight single-scatter-simulation (TOF-SSS) algorithm [1]. However, the available implementations are vendor-specific and proprietary, therefore not openly available for research purposes. Moreover, they only work for the specific geometry of the corresponding clinical scanner. Open-source reconstruction libraries exist, such as CASToR (Customizable and Advanced Software for Tomographic Reconstruction) [2] and STIR (Software for Tomographic Image Reconstruction) [3], but they do not offer scatter estimation for TOF and custom geometries. Alternatively, open-source Monte-Carlo physics simulation software, such as GATE [4], can be used to estimate scatter contributions during reconstruction. Although this offers full flexibility and configuration with the most accuracy [5, 6], computation times are long and therefore, not suited for prototyping, extensive studies or embedded application on real systems.

The accurate estimation of scatters is commonly performed with prior information on the attenuation properties of the scanned object, usually obtained from a CT scan. This may not always be possible though, as it is the case for dedicated PET-only or PET-MRI systems. Nonetheless, the typical TOF-SSS algorithm may still be applied in these scenarios through joint activity and attenuation reconstruction [7], although dedicated scatter estimation methods have also been proposed, using only TOF and energy information [8] or using Machine-Learning [9].

In this article, we present openSSS: an open-source implementation of the 3D single-scatter-simulation algorithm with TOF. openSSS expands on the original TOF-SSS algorithm by introducing scaling of the scatter distribution in the image domain, rather than in the sinogram domain. Moreover, the interpolation of the scatter distribution is performed for all (allowed) ring differences, rather than calculated separately for each ring difference or approximated through sinogram rebinning. It is fully configurable with support for custom geometries and detector setups, and it is compatible with open-source reconstruction libraries, such as CASToR and STIR. This study evaluates openSSS for three different scanner geometries, on NEMA phantoms and patient data, and compares the results against three different software packages: (1) GATE Monte-Carlo simulations, (2) Philips’ research reconstruction platform PURE and (3) Siemens Healthineers’ prototype reconstruction software e7tools. openSSS is publicly available for download in [10].

## Methods

### Coincidence definition

In the context of this work, prompts are defined as the total measured coincidences, including random and scattered events. Randoms represent coincidences from different annihilation events and from noise in the PET system. In contrast, true coincidences originate from the same annihilation event without scattering. These classifications are purely abstract, as it is not possible to distinguish and filter the measured coincidences a priori. Following this reasoning, net trues are defined as measured prompts minus the estimation of randoms.

### Single scatter simulation

#### Estimation

**Fig. 1:**
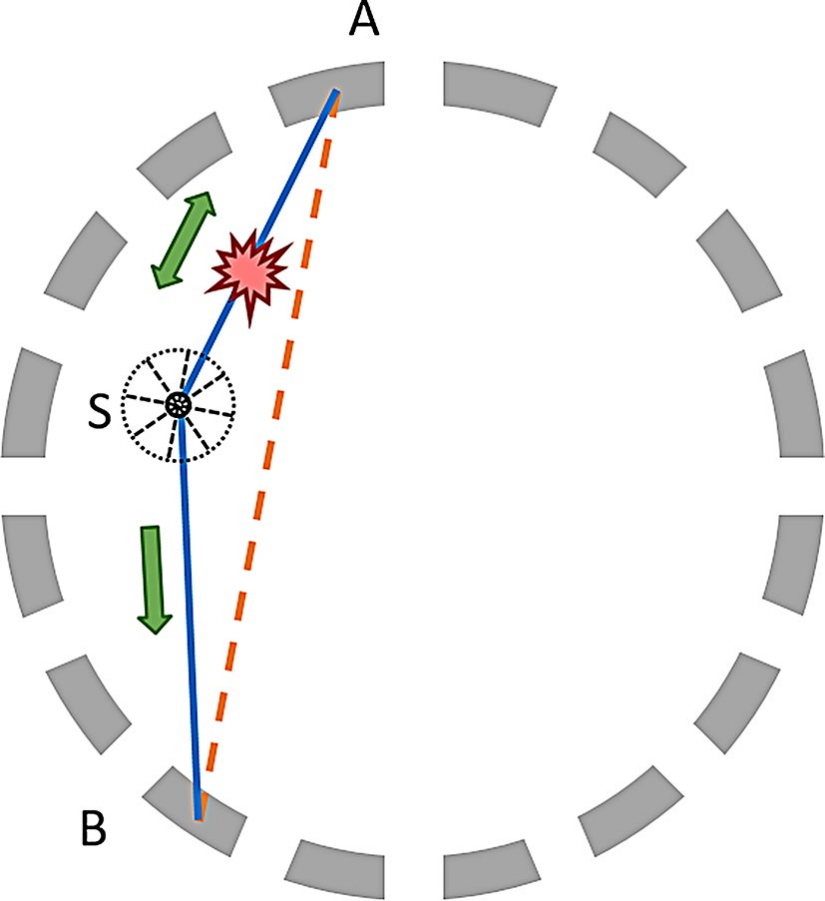
Scattering example for a pair of detectors A-B and a scatter point S. Photons scatter in a spherical distribution, as represented in the figure, with probabilities following the Klein-Nishina formula. For this setup, emission events closer to A contribute with scattered photons towards B and vice-versa. The real photons’ path (solid line) is significantly shifted from the LOR (ticked line), which illustrates the need for scatter correction for quantitative PET image reconstruction

The single-scatter-simulation (SSS) algorithm for TOF is based on a physical photon model of Compton scattering and the specific detectors’ geometry in the system [1], as illustrated in Fig. 1. It only considers scattering of one annihilation photon and only once (single scattering).

Given a pair of detectors A-B and a scatter point S, the scatter probability depends on three major components: how many photons are expected to reach the scatter point S, how many photons scatter with angle ASB and how many photons reach the detectors. The first and last components are a function of the activity and attenuation on the photon’s path (AS and BS). TOF information provides more precision on the location of the emission point, splitting the ASB line into segments. The second component is known as the differential cross-section and it is modeled by the Klein-Nishina formula ($\:d\sigma\:/d{\Omega\:}$):

$$\:\frac{d\sigma\:}{d{\Omega\:}}=\frac{1}{2}{r}_{e}^{2}{\left(\frac{\lambda\:}{{\lambda\:}^{{\prime\:}}}\right)}^{2}\left[\frac{\lambda\:}{{\lambda\:}^{{\prime\:}}}+\frac{{\lambda\:}^{{\prime\:}}}{\lambda\:}-{\text{sin}}^{2}\left(\theta\:\right)\right]\text{with}\frac{\lambda\:}{{\lambda\:}^{{\prime\:}}}=\frac{{E}_{{\lambda\:}^{{\prime\:}}}}{{E}_{\lambda\:}}=\frac{1}{1+\frac{{E}_{\lambda\:}}{{m}_{e}{c}^{2}}(1-\text{cos}\left(\theta\:\right))}$$

where $\:{r}_{e}$ is the classical electron radius, $\:\lambda\:$ and $\:{\lambda\:}^{{\prime\:}}$ the incident and scattered photons with $\:{E}_{\lambda\:}$ and $\:{E}_{{\lambda\:}^{{\prime\:}}}$ their energy respectively, $\:\theta\:$ the scattering angle, $\:{m}_{e}$ the electron rest mass and $\:c$ the speed of light.

Scatter points were sampled from the whole attenuation map, including outside the field of view. Sampling in the axial direction of the scanner is limited to 1.5 times the FOV, from the center of the scanner and in both direction. Probabilities were estimated for each combination of detector pair (LOR) and scatter point, based on the three components referenced in the previous paragraph. For each detector pair (LOR), the estimated (single) scatter probability for each TOF bin is equal to the sum of the (single) scatter probabilities for all the scatter points on the corresponding TOF bin.

#### Interpolation

Interpolation was implemented in openSSS, to calculate the complete scatter distribution from a small sample of LORs and scatter points, estimated from downsampled activity and attenuation maps. This implementation expands on the standard SSS [1] by interpolating the scatter estimate for detector pairs in different rings, to populate oblique sinograms of all the (allowed) ring differences. The number and sampling scheme for LORs and scatter points is fully customizable, with the current implementation using a uniform sampling based on previous studies [11].

Interpolation is directly performed in the complete sinogram space, i.e. without a sinogram rebinning step. Interpolation uses a gridded approach, by taking the sinogram coordinates as a mesh grid, on which the sampled LORs correspond to sampled grid points. The data space is interpreted as four dimensional, given by: relative projection angle, relative distance from ring center, relative axial distance of first ring and relative axial distance of second ring.

To optimize interpolation, the coordinates of grid points are considered unitary, so that the distance between consecutive LORs in sinogram space is always one in every axis. Each grid point in the sinogram space still has correspondence to the unique crystal pair and the spatial coordinates that compose that LOR, so there is no loss of information. However, only the unit grid coordinates are used for interpolation. As this simplifies the data space, computation times are minimized but it may also introduce interpolation errors for systems with very irregular geometries, depending on the sampling scheme for LORs. The more the grid-points and the better the sampling scheme, the more accurate the scatter estimation.

#### Scaling

The abovementioned model-based scatter estimation gives the relative single-scatter probability for every LOR. Subsequently, to include multiple scatters, quantitative scaling of the scatter distribution was performed. This is based on the assumption that the addition of multiple scatters does not alter the shape of the scatter distribution but only the height. It is often referred to as tail fitting, as it uses the tails of the scatter distribution outside of the body/phantom contour (where no unscattered true coincidences are expected in the measured data) to determine the ratio of scatters vs. unscattered true coincidences.

**Fig. 2:**
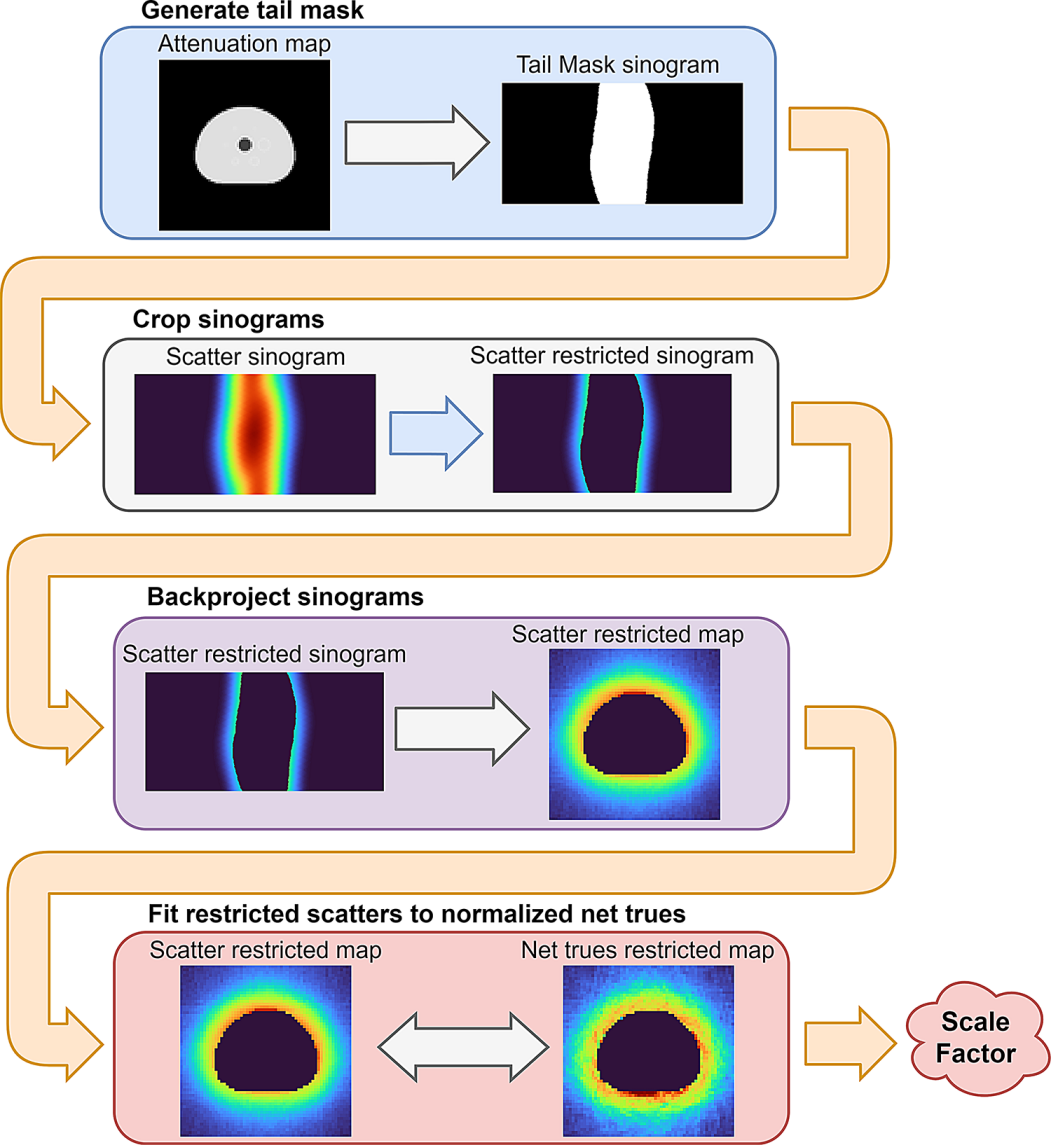
Step by step pipeline of the scatter scaling to the normalized measured net trues, highlighting the image-domain approach implemented for openSSS. Due to the sparse nature of the measured data, the backprojected image of the masked net trues is less smooth than the one for the estimated scatters, as observed

The scaling method directly affects the goodness of the scatter correction, and it is a trade-off between simplicity, accuracy and reliability. Our scaling implementation deviates from the originally proposed scaling [1] by being performed in the image domain, through simple (unfiltered) backprojection of the tails-restricted normalized measured net trues and of the estimated scatters. The pipeline is illustrated in Fig. 2. This approach is chosen as it is independent of the scanner geometry and of the sinogram definition. The image domain also provides stability on the scaling for sparse sinograms and with small number of measured counts per LOR. The spatial distribution of the tails-restricted scatters is fitted to the spatial distribution of the measured net trues, through the linear least-square method. Comparison is performed voxel by voxel, so that a single global factor is obtained, to be directly applied on scaling the scatter sinograms.

#### Framework

**Fig. 3:**
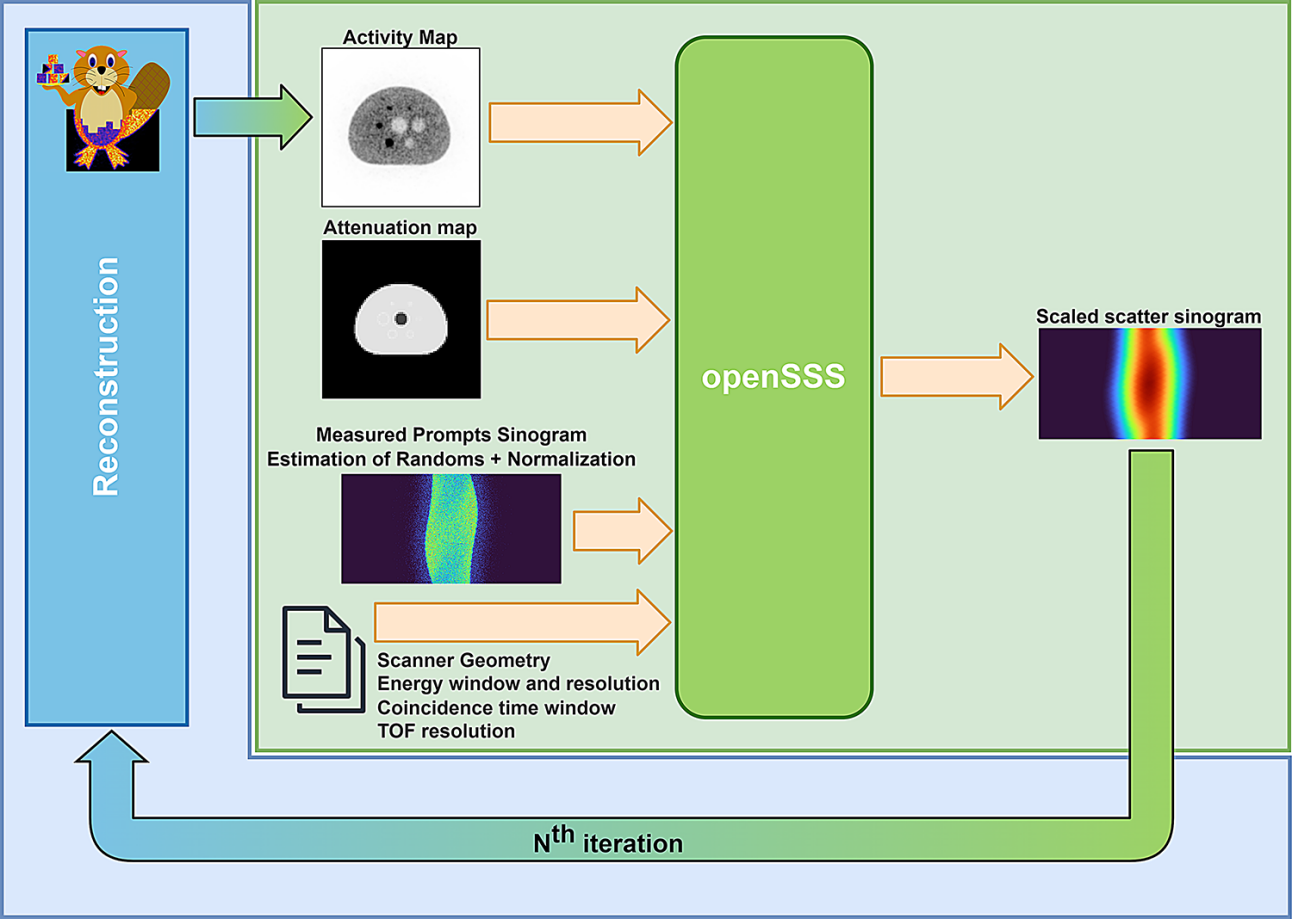
Workflow of openSSS (green block) integrated with PET reconstruction libraries (blue block). The process is iterative, as the scatter sinograms are used to estimate a new activity map, which can be used to estimate new scatter sinograms

openSSS was developed to provide scatter correction during reconstruction, following the workflow illustrated in Fig. 3. It takes as input the measured prompts sinogram (TOF-binned), the attenuation map and the current estimation of the activity map, to be updated sequentially after image reconstruction. Information on the PET system is required, namely on the geometry (coordinates of each detector) and specifications (energy resolution, energy window, TOF resolution, coincidence timing window). These parameters define the scatter paths and detection efficiency, according to the original TOF-SSS algorithm [1]. In order to scale the scatter distribution, estimation of randoms and normalization correction factors are applied to the measured prompts, to obtain the normalized measured net trues. No scatter correction is performed on the first step.

In this work, we used the CASToR reconstruction library to perform image reconstruction with the scatter estimations from openSSS. To this end, we developed interface bridges between the two libraries: one to read the datafiles and export them to sinograms, and another to read the scatter correction factors and add them to the datafiles.

### Geometrical setups

Three different PET system geometries were used for validation: two clinical commercial systems - Biograph Vision 600 (Siemens Healthineers) and Biograph mCT (Siemens Healthineers) - and the dedicated UMC Utrecht radiotherapy PET/MRI [12] (wide-bore Philips Ingenia 1.5T MRI with PET detectors placed in the gap of the split-gradient coil). The scanners were integrated into openSSS through a parameter file, composed of the coordinates of every crystal of the system and the scanner specifications on energy and timing. The reference values used are as following: 10.8% energy resolution, 435 keV energy threshold, 264ps TOF resolution and 1000ps TOF range for the Biograph Vision 600 (Siemens Healthineers); 14.5% energy resolution, 435 keV energy threshold, 580ps TOF resolution and 2028ps TOF range for the Biograph mCT (Siemens Healthineers); 10.8% energy resolution, 435 keV energy threshold, 264ps TOF resolution and 1000ps TOF range for the UMC Utrecht PET/MRI.

### OpenSSS performance evaluation

The validation of openSSS was based on three different sets of experiments, as summarized in Table 1.

**Table 1:** Summary of the experiments to validate the performance of opensss, highlighting the origin, type and object of the dataset, the scanner, the reconstruction framework utilized and the scatter fraction (for the simulated datasets)

Experiment	Dataset	Geometry	Object	Reconstruction	Scatter fraction
A	Simulated (list-mode)	Biograph Vision 600 (Siemens Healthineers)	NEMA phantom	CASToR (list-mode)	33%
B	Simulated (list-mode)	UMC Utrecht PET/MRI	NEMA phantom	CASToR & PURE (list-mode)	40%
C	Clinical patient data (sinogram)	Biograph mCT (Siemens Healthineers)	Pelvis scan Head and neck scan	CASToR (sinogram)	

Experiment (A) compared reconstructions of openSSS-derived scatter correction with unscattered true coincidences from Monte-Carlo simulations. Experiment (B) compared reconstructions of openSSS-derived scatter correction with Monte-Carlo-derived scatter correction from Philips’ research reconstruction platform PURE. Experiment (C) compared reconstructions of openSSS-derived scatter correction with clinical-derived scatter correction from Siemens Healthineers’ prototype research reconstruction platform e7tools.

#### GATE Monte-Carlo simulations

Monte-Carlo simulations offer a ground-truth reference. The simulated coincidences can be sorted by type, enabling the removal of random and scatter coincidences to obtain true coincidences, for a focused evaluation of only the scatter estimation. The reconstructed image with scatters corrected by openSSS was benchmarked on image-quality against reconstruction of only unscattered true coincidences (without scatters or randoms). GATE was used for simulation of the NEMA phantom on the Biograph Vision 600 (Siemens Healthineers) system. CASToR was used for reconstruction following the NEMA NU 2-2007 protocol [13], comparing results on contrast recovery, background variability and residual error.

The NEMA IEC Body Phantom [13] was used, with a background activity of 5MBq/ml and background to hot sphere contrast of 1:4. The largest two spheres (diameter 28 mm and 37 mm) were left without activity. The middle cylinder was filled with lung-like material (density 0.30 g/cm3). The phantom was placed in the center of the system and for statistical significance, simulated acquisitions targeted a total number of prompts events in the order of hundred millions, saved in list-mode and with continuous TOF information. Simulations were performed with these settings for the Biograph Vision 600.

#### Philips’ research platform ‘PURE’

Monte-Carlo simulations can also be used exclusively for estimation of scatters, based on reconstructed activity distributions and attenuation maps from acquired transmission data [14]. With this approach, instead of estimating scatters with the SSS algorithm, isolated Monte-Carlo simulations are performed in alternation with the iterative image reconstruction process. Philips’ research platform ‘PURE’ operates this way: it first reconstructs an image without scatter correction, which is then used as a reference activity map for a first Monte-Carlo simulation, from which a first estimation of scatters is obtained. This scatter distribution is used in a second reconstruction, repeating the cycle.

openSSS was compared to PURE for evaluation of the complete framework. GATE was used to simulate the NEMA phantom on the dedicated UMC Utrecht radiotherapy PET/MRI and using the same settings as experiment A), filtering out random coincidences. Data was reconstructed with PURE using the proprietary scatter correction algorithm [15] and compared to openSSS correction reconstructed with CASToR. Similarly to openSSS, PURE was configured to also perform scaling of scatters in the image domain. Image quality was evaluated on contrast recovery, background variability and residual error (see Supplementary Materials).

#### Siemens Healthineers’ proprietary reconstruction software

For evaluation of the real-world performance of openSSS, patient data was reconstructed with our proposed framework and compared with Siemens Healthineers’ proprietary reconstruction software. It is also based on the TOF-SSS algorithm but performs scaling of scatters in the sinogram domain. The Siemens Healthineers’ software was only used to process the proprietary datafiles from the scanner and to calculate a reference scatter distribution. This scatter estimation was used for reconstruction with CASToR and compared to openSSS correction, also reconstructed with CASToR.

Additionally, clinical data acquired on the Biograph mCT (Siemens Healthineers) from two patients was used; a head and neck scan and a pelvis scan. A single bed position was reconstructed for evaluation. The data was saved in sinogram format, with binned TOF (13 bins) and mashed LORs (11 axial and 2 angular), according to the vendor’s specifications. As the clinical data used was anonymized, written informed consent was waived by our medical ethical committee.

### NEMA phantom evaluation

**Fig. 4:**
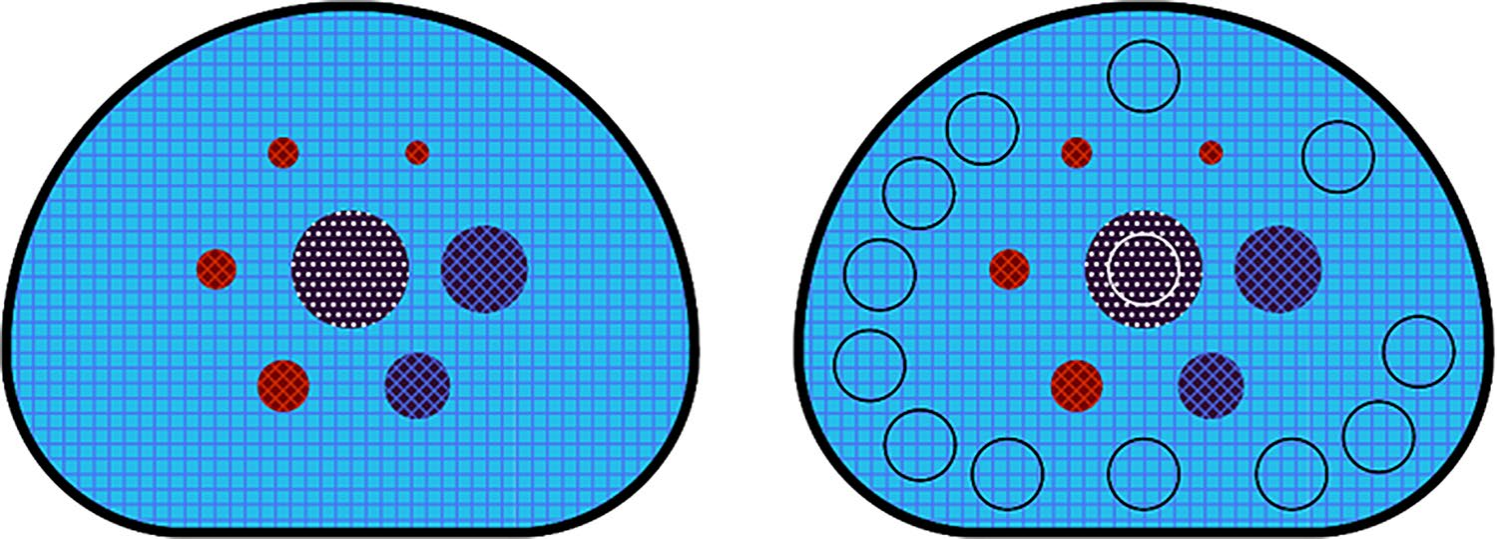
NEMA phantom as specified by the protocol, with 1:4 background to hot sphere contrast, the two larger spheres without activity and the middle insert with lung-like material. On the right, a set of circular ROIs is identified for the largest sphere, to illustrate the procedure to calculate the metrics for image quality evaluation, as defined in the NEMA protocol

Evaluation of the simulated results on the NEMA IEC body phantom followed the NEMA NU 2-2007 protocol [13], comparing results on contrast recovery, background variability and residual error. Circular Regions of Interest (ROI) were drawn for each sphere, with radius matching the corresponding sphere size, one at the sphere location (cross pattern circles) and twelve in the background (black circles, drawn for the largest sphere), as exemplified on Fig. 4.

Contrast recovery measures the contrast characteristics of the hot and cold spheres in the warm background and provides an indication of lesion detection in clinical applications. For each hot sphere $\:j$, the contrast recovery $\:{Q}_{H,j}$ is defined as:

$$\:{Q}_{H,j}\:=\:\left(\frac{{c}_{H,j}}{{c}_{B,j}}-1\right)\:/\:\left(\frac{{a}_{H}}{{a}_{B}}-1\right)\times\:100\%$$

Where $\:{c}_{H,j}$ is the mean value in the ROI for the hot sphere $\:j$, $\:{c}_{B,j}$ is the mean value of the background ROIs for sphere $\:j$, while $\:{a}_{H}$ and $\:{a}_{B}$ are the activity concentration in the hot sphere and background respectively.

For the cold sphere *j*, the contrast recovery $\:{Q}_{B,j}$ is defined as:

$$\:{Q}_{H,j}\:=\:\left(1-\frac{{c}_{C,j}}{{c}_{B,j}}\right)\times\:100\%$$

Where $\:{c}_{C,j}$ is the mean value in the ROI for the cold sphere $\:j$ and $\:{c}_{B,j}$ is the mean value of the background ROIs for sphere $\:j$.

The background variability measures the uniformity of the warm background and for sphere $\:j$ it is defined as:

$$\:{N}_{j}=\left(\frac{S{D}_{j}}{{C}_{B,j}}\right)\cdot\:100\%$$

Where $\:S{D}_{j}$ is the standard deviation of the values in the background ROIs of sphere $\:j$.

The residual error is measured in a cold region and measures the accuracy of the attenuation and scatter correction. For each slice $\:i$, the residual error $\:\varDelta\:{c}_{lung,i}$ is defined as:

$$\:{{\Delta\:}}_{lung,i}=\frac{{c}_{lung,i}}{{c}_{B,37\:mm}}\times\:100\%$$

Where $\:{c}_{lung,i}$ is the mean value in the lung insert ROI at slice $\:i$ (white circle in Fig. 4) and ${C}_{B,\,37mm}$is the mean value in the background ROIs for the cold sphere with radius 37 mm (black circles in Fig. 4).

### Patient data evaluation

To evaluate the performance of openSSS on clinical patient data, the reconstructed images are compared visually and through line profiles. To quantify deviations, the mean voxel intensity at ROIs is calculated and compared on divergence and ratio. These ROIs are defined based on 3D isocontours at regions with hot lesions that stand out from the background.

### Framework settings

Image reconstruction was performed using CASToR. The 3D ordered subset expectation maximization algorithm (OSEM) was used with the same settings for all reconstructions, based on indicative guidelines [16]: 3 iterations and 17 subsets (product of iterations and subsets larger than 50), an isotropic voxel size of 2 mm and a Gaussian post-reconstruction filter with an isotropic kernel of 5 mm FWHM. Point-spread-function modelling was not performed in the reconstruction. Scatter correction is performed within OSEM in the forward projection step. In experiment B), the images reconstructed through Philips’ PURE also used OSEM with the same settings described above.

A new scatter estimate was calculated after each reconstruction iteration for all scatter estimation algorithms, meaning two scatter distributions are calculated per reconstruction. openSSS was fully configured to use activity and attenuation maps cropped in the axial direction (Z axis) to the axial length of the scanner and downsampled by an isotropic factor of 3. Scatter points were sampled every 3 voxels in the X and Y direction and every 2 voxels in the Z direction, skipping voxels with zero local attenuation coefficient. The number of sampled detectors was set to ¼ of the total number and the number of rings to 6 [11], whereas TOF information was aggregated into 13 bins. As for Philips’ and Siemens Healthineers’ proprietary software, settings are locked to optimal values chosen by the vendor.

openSSS was implemented in Matlab and tested on version 2021.a, with the calculations performed on a desktop computer with a 32-core 3.6 GHz Ryzen Threadripper and 128GB of RAM. This implementation was parallelized at the scatter point level, with each parallel process computing the scatter probability at that point for all sampled LORs. A total of 12 simultaneous parallel processes were utilized, based on a balance between the geometry of the system and computational resources.

## Results

### GATE Monte-Carlo simulations

**Fig. 5:**
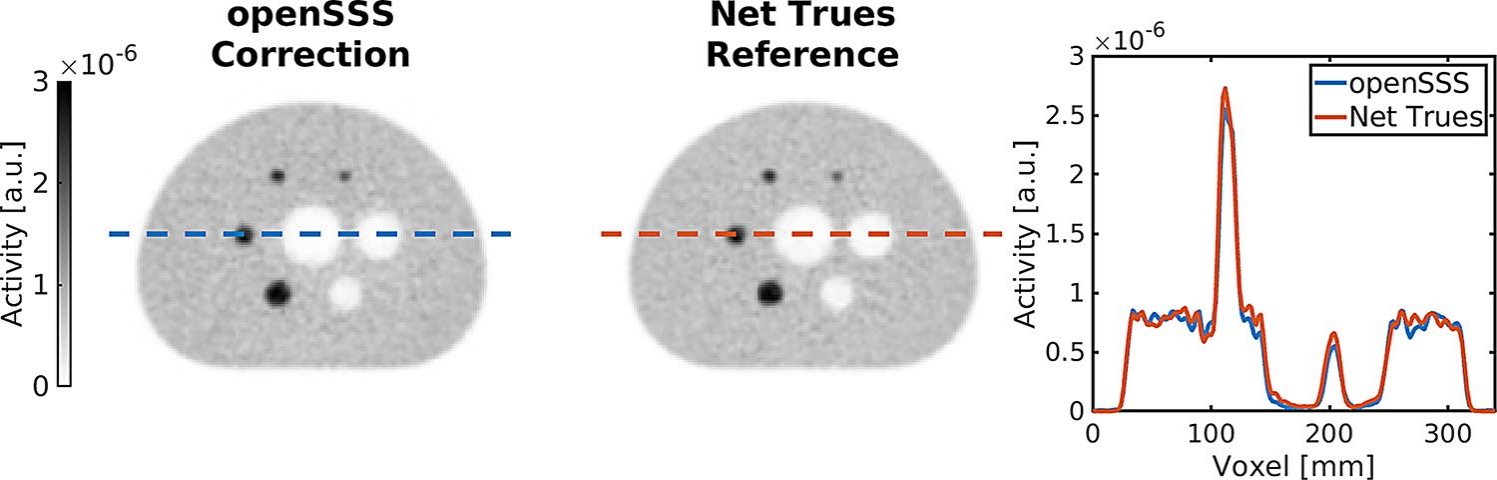
PET images with line profiles for Method A - NEMA phantom simulated on the Biography Vision 600 (Siemens Healthineers) - through the central slice intersecting the center of the spheres, reconstructed with scatters corrected with openSSS and reconstructed with net trues (unscattered and nonrandom coincidences)

Reconstructed images for the GATE simulated NEMA phantom on the Biograph Vision 600 (Siemens Healthineers) scanner geometry are shown in Fig. 5, for the openSSS scatter correction and for the net trues reference (unscattered and nonrandom coincidences only). The images are comparably equivalent, as highlighted by the line profile, with contrast deviating by a mean of 2.4%-point, while background uniformity differ by 0.4%-point. As such, the images have an equally uniform background and well-defined contrasting spheres, both in cold and hot regions. The full image quality metrics (see also Supplementary Materials) are reported in Table 2.

**Table 2:** Image quality metrics for method A - NEMA Phantom simulated on the biograph vision 600 (Siemens Healthineers) - reconstructed with scatters corrected with opensss and reconstructed with net trues (reference: unscattered non-random coincidences)

System	Sphere (mm)	Contrast recovery (%)		Background variability (%)		Residual error (%)	
		*openSSS*	*Reference*	*openSSS*	*Reference*	*openSSS*	*Reference*
Biograph Vision 600 (Siemens Healthineers)	10	33	30	5	5	5	8
	13	43	43	4	4		
	17	49	53	3	3		
	22	66	69	3	2		
	28	69	71	3	2		
	37	81	79	2	2		

### Philips’ research platform ‘PURE’

**Fig. 6:**
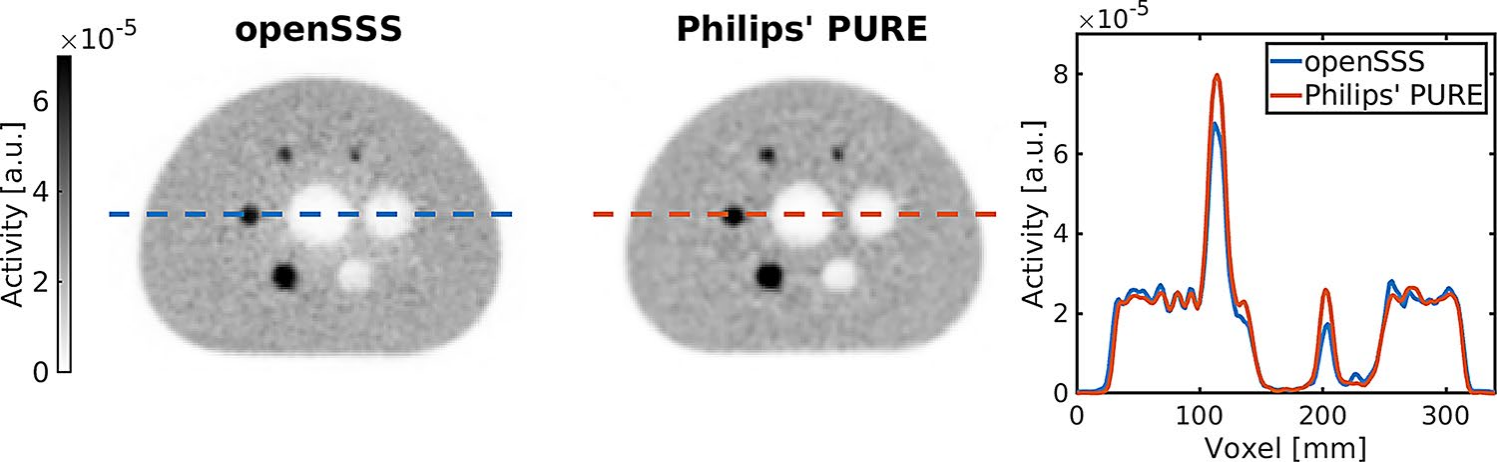
PET images with line profiles for Method B - simulated NEMA phantom on the geometry of the UMC Utrecht radiotherapy PET/MRI - through the central slice intersecting the center of the spheres, reconstructed with scatters corrected with openSSS and corrected with Philips’ PURE

Reconstructed images using openSSS and using Philips' PURE scatter estimation are shown in Fig. 6, on the UMC Utrecht radiotherapy PET/MRI scanner geometry. The images are visually similar, without any visible scatter artifacts in the cold spots and with a mean of 1.8%-point for the background variability divergence. There are differences between the activity distributions, as contrast deviates by a mean of 3.7%-point, mostly from differences at the smallest of the hot spheres. It is also noticeable in the background, between the center and the outer regions as highlighted by the deviation of 2.8%-point in the residual error. For the openSSS scatter estimation, the center region around the cold insert is observed less intense than at the borders of the phantom. This is better observed when comparing the line profiles. Metrics on the image quality are presented in Table 3.

**Table 3:** Image quality metrics for method B - simulated NEMA Phantom on the geometry of the UMC Utrecht radiotherapy PET/MRI - reconstructed with scatters corrected with opensss and corrected with Philips’ PURE

System	Sphere (mm)	Contrast recovery (%)		Background variability (%)		Residual error (%)	
		*openSSS*	*PURE*	*openSSS*	*PURE*	*openSSS*	*PURE*
UMC Utrecht radiotherapy PET/MRI	10	34	30	5	9	8	5
	13	34	36	4	7		
	17	42	49	4	5		
	22	54	64	3	5		
	28	66	66	3	4		
	37	75	75	3	3		

### Siemens Healthineers’ prototype reconstruction platform

**Fig. 7:**
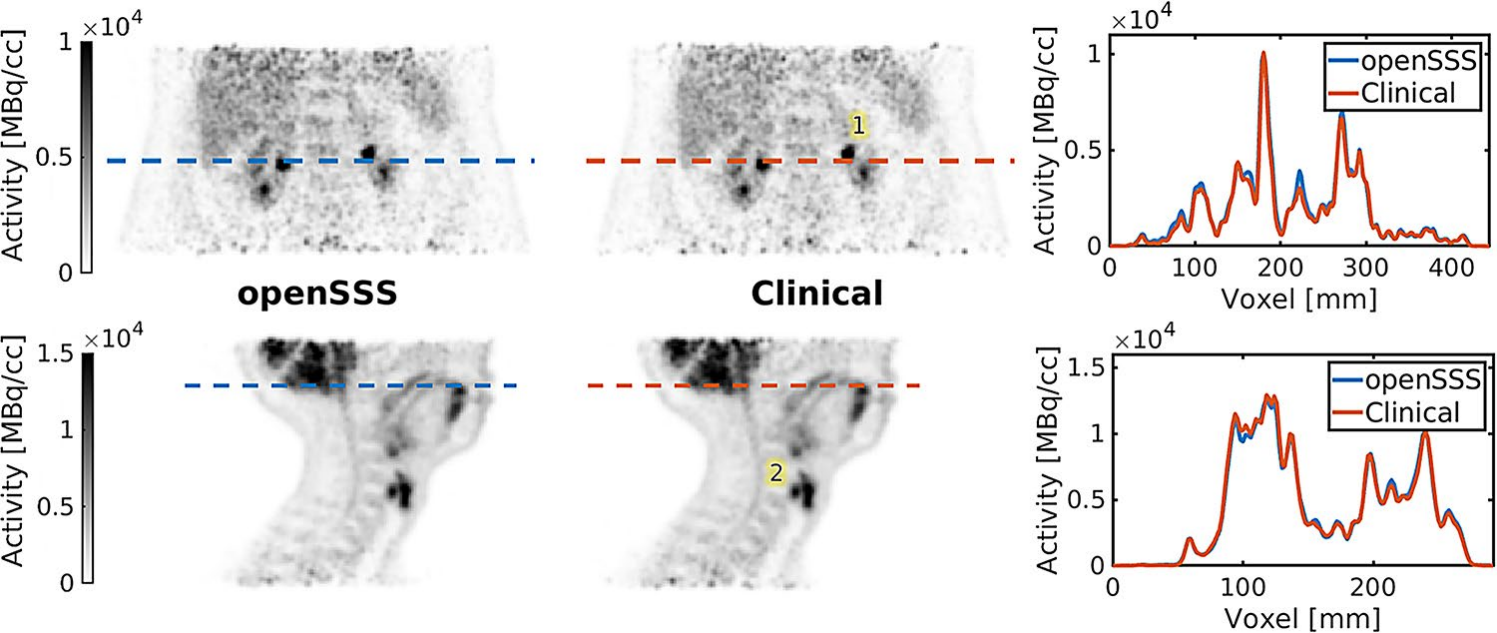
PET images with line profiles for Method C - clinical patient data acquired in the Biograph mCT (Siemens Healthineers) scanner - reconstructed with scatters corrected with openSSS and corrected with Siemens Healthineers’ e7tools, on two body regions of two patients. ROIs have been delineated for each patient, identified with numbers on the reconstructed images

Clinical data reconstructed with scatters estimated using openSSS and using Siemens Healthineers’ software is shown in Fig. 7, acquired at the Biograph mCT (Siemens Healthineers) clinical scanner and reconstructed with CASToR on the same settings. The images are visually equivalent, with similar levels of noise and without any streak artifacts or activity outside the patients’ contour. Regions with higher activity are equally contrasted in detail, without overestimation of scatters around them. The hot lesions are surrounded by uniform activity without a colder halo and at the ROIs, the mean divergence is 1.8%-point for the mean intensity. The separation between vertebra is also observed to be preserved. Metrics for the ROIs are presented in Table 4.

**Table 4:** Image quality metrics for method C - clinical patient data acquired in the biograph mCT (Siemens Healthineers) scanner - reconstructed with scatters corrected with opensss and corrected with Siemens healthineers’ e7tools

Patient	Mean intensity (MBq/cc)		Divergence (%)	Ratio
	openSSS	Clinical		
1	8086	7903	2.3	1.02
2	15,463	15,643	1.2	0.99

## Discussion

In this article, we have developed and validated an open-source implementation of the TOF-aware SSS algorithm for scatter correction. The reconstructed images and the metrics deviate by up to 3.7%-point on contrast recovery and 1.8 on background variability, showing good agreement with reference simulated data and two vendor-specific research platforms, on three scanner geometries. These results were obtained without extensive optimization or fine-tuning of the settings of openSSS for each experiment and scanner, validating the general compatibility of the framework.

The figures and tables validate our implementation of the TOF-SSS algorithm, namely on the estimation, interpolation and scaling. A direct evaluation and comparison of each component is not performed, due to challenges inherent to the closed nature of the vendors’ platforms. Nonetheless, as the images with openSSS are similar to those obtained through the vendors’ platforms, on image quality and metrics, it is demonstrated that all the components are correctly implemented. Furthermore, experiment C) against Siemens Healthineers’ platform represents a comparison with a clinically-validated SSS implementation with CE/FDA clearance, demonstrating that openSSS may allow for clinical grade reconstructions.

openSSS performs scaling in the image domain and not by direct comparison of the sinograms. This bypasses the sparsity and small dynamic range of sinograms obtained for short acquisitions and on systems with gaps and/or large planar detector blocks, which may limit the goodness of the fit. To this end, the experiments were all performed for long acquisitions as to guarantee reliable statistics (high number of counts), in accordance with clinical practices and protocols [16]. The results show appropriate scaling of the estimated scatter distributions, specially supported by the simulation experiment A) on the comparison against reconstructed NEMA data of only net true coincidences.

The possibility of applying openSSS to custom PET geometries is further validated by experiment B) on the UMC Utrecht radiotherapy PET/MRI. This scanner is composed of planar detector blocks with gaps in between, oriented at different angles around the iso-center, which is not directly accounted for in our implementation, namely at the interpolation level of the scatter estimation. More precisely, we simplified the data space so that any pair of consecutive LORs is one unit apart in every axis. For irregular geometries, such as those with large gaps between detector blocks, there can be jumps in the angle of the LORs and on their distance to the center, which makes the approximation to the unit grid model less accurate. In interpolation, only these simplified sinogram grid coordinates are used, so errors propagate. Nonetheless, the metrics support a balanced scatter estimation by openSSS for the irregular geometry of the UMC Utrecht radiotherapy PET/MRI, as shown in Table 3. The results could potentially be improved though, as more accurate results are observed for the more regular geometry of the Biograph Vison 600 (Siemens Healthineers), with less deviations from the reference. That could be achieved, for example, through use of optimized sampling schemes for the LORs.

In estimating scatters, we have fixed the parameters of openSSS for all experiments (sampling scheme of scatter points and detectors, sampling of activity and attenuation maps, number of TOF bins), chosen based on literature but not optimized for the specific geometry or data. We have assumed these values to correspond to a general balance between estimation accuracy and computation time. Results could therefore be improved through specific configuration of the openSSS parameters, optimized for the specific geometry of the scanner and the data characteristics. The rationale is that more accurate results can be obtained with more scatter points, more detectors, more TOF bins and higher resolution for the activity and attenuation maps, at the expense of more memory requirements and longer computation time. However, the improvement in accuracy may be minimal and consequently not worth the computational burden. Such fine-tuning is performed by vendors before embedding their proprietary SSS implementations, offering optimal results but little to no control to the user on the scatter estimation settings in the clinical reconstruction software.

## Conclusion

With openSSS, we have developed and validated a 3D TOF-aware scatter estimation library for PET reconstruction. openSSS enables reconstructions of clinical quality, essential for quantitative PET. It improves and elevates open-source reconstruction libraries, such as CASToR and STIR, by allowing transparent research with vendor-independent software, with complete insight and transparency on the methods and with full control of the parameters. It is also compatible with custom scanner geometries, enabling prototyping of novel PET scanners.

## Data Availability

The framework presented in the study is openly available online, hosted on GitHub (see References section). The datasets used and analyzed during the current study are available from the corresponding author on reasonable request.

## References

[1] Watson CC. Extension of single scatter simulation to scatter correction of time-of-flight PET. IEEE Nuclear Science Symposium Conference Record. 2005; Fajardo, PR, USA, p. 2492–2496. 10.1109/NSSMIC.2005.1596846

[2] Merlin T, Stute S, Benoit D, Bert J, Carlier T, Comtat C. CASToR: a generic data organization and processing code framework for multi-modal and multi-dimensional tomographic reconstruction. Phys Med Biol. 2018 Sep;63(18). 10.1088/1361-6560/aadac1. 10.1088/1361-6560/aadac1 30113313

[3] Thielemans K, Tsoumpas C, Mustafovic S, Beisel T, Aguiar P, Jacobson MW, et al. STIR: Software for tomographic image reconstruction release 2. Phys Med Biol. 2012 Jan;57(4):867-83. 10.1088/0031-9155/57/4/867. 10.1088/0031-9155/57/4/867 22290410

[4] Jan S, Santin G, Strul D, Staelens S, Assié K, Autret D, et al. GATE: a simulation toolkit for PET and SPECT. Phys Med Biol. 2004 Oct;49(19):007. 10.1088/0031-9155/49/19/007. 10.1088/0031-9155/49/19/007 PMC3267383 15552416

[5] Zaidi H. Comparative evaluation of scatter correction techniques in 3D positron emission tomography. Eur J Nucl Med. 2000;27:1813–26. 10.1007/s002590000385. 11189945 10.1007/s002590000385

[6] Polycarpou I, Thielemans K, Manjeshwar R, Aguiar P, Marsden PK, Tsoumpas C. Comparative evaluation of scatter correction in 3D PET using different scatter-level approximations. Ann Nucl Med. 2011;25:643–9. 10.1007/s12149-011-0514-y. 21751085 10.1007/s12149-011-0514-y

[7] Rezaei A, Schramm G, Willekens SMA, Delso G, Laere KV, Nuyts J. A quantitative evaluation of joint activity and Attenuation reconstruction in TOF PET/MRI brain imaging. J Nucl Med. 2019;60(11):1649–55. 10.2967/jnumed.118.220871. 30979823 10.2967/jnumed.118.220871 PMC6836858

[8] Álvarez-Gómez JM, Santos-Blasco J, Moliner Martínez L, Rodríguez-Álvarez MJ. Fast energy dependent scatter correction for List-Mode PET. Data J Imaging. 2021 Sep;7(10):199. 10.3390/jimaging7100199. 10.3390/jimaging7100199 PMC8541469 34677285

[9] Jahangir R, Kamali-Asl A, Arabi H, Zaidi H. Strategies for deep learning-based Attenuation and scatter correction of brain ^18^F-FDG PET images in the image domain. Med Phys. 2024 Jan;51:870-80. 10.1002/mp.16914 38197492

[10] GitHub. openSSS [Internet]. Available from: https://github.com/r-santo/openSSS

[11] Accorsi R, Adam LE, Werner ME, Karp JS. Optimization of a fully 3D single scatter simulation algorithm for 3D PET. Phys Med Biol. 2004 Jun;49(12):008. 10.1088/0031-9155/49/12/008. 10.1088/0031-9155/49/12/008 15272675

[12] Branderhorst W, Steensma BR, Beijst C, Huijing ER, Alborahal C, Versteeg E, et al. Evaluation of the radiofrequency performance of a wide-bore 1.5 T positron emission tomography/magnetic resonance imaging body coil for radiotherapy planning. Phys Imaging Radiat Oncol. 2021;17:13–9. 10.1016/j.phro.2020.12.002. 33898772 10.1016/j.phro.2020.12.002 PMC8057958

[13] National Electrical Manufacturers Association. Performance measurements of positron emission tomographs NEMA standards publication NU 2-2007. Rosslyn, VA: National Electrical Manufacturers Association; 2007.

[14] Levin CS, Dahlbom M, Hoffman EJ. A Monte Carlo correction for the effect of Compton scattering in 3-D PET brain imaging. IEEE Trans Nucl Sci. 1995 Aug;42(4):467-80. 10.1109/23.467880.

[15] Salomon A, Goedicke A, Schweizer B, Aach T, Schulz V. Simultaneous reconstruction of activity and Attenuation for PET/MR. IEEE Trans Med Imaging. 2011 Mar;30(3):804-13. 10.1109/tmi.2010.2095464. 10.1109/TMI.2010.2095464 21118768

[16] Boellaard R, O’Doherty MJ, Weber WA, Mottaghy FM, Lonsdale MN, Stroobants SG, et al. FDG PET and PET/CT: EANM procedure guidelines for tumour PET imaging: version 1.0. Eur J Nucl Med Mol Imaging. 2010;37:181–200. 10.1007/s00259-014-2961-x. 19915839 10.1007/s00259-009-1297-4 PMC2791475

